# Eumelanin Precursor 2-Carboxy-5,6-Dihydroxyindole (DHICA) as Doping Factor in Ternary (PEDOT:PSS/Eumelanin) Thin Films for Conductivity Enhancement

**DOI:** 10.3390/ma13092108

**Published:** 2020-05-02

**Authors:** Ludovico Migliaccio, Felice Gesuele, Paola Manini, Maria Grazia Maglione, Paolo Tassini, Alessandro Pezzella

**Affiliations:** 1 Department of Chemical Sciences, University of Naples “Federico II”, 80126 Naples, Italy; ludovico.migliaccio@unina.it (L.M.); paola.manini@unina.it (P.M.); 2 Department of Physics “Ettore Pancini”, University of Naples “Federico II”, 80126 Naples, Italy; felice.gesuele@unina.it; 3 Laboratory for Nanomaterials and Devices (SSPT-PROMAS-NANO), ENEA C. R. Portici, Piazzale Enrico Fermi 1, Località Granatello, 80055 Portici, Italy; mariagrazia.maglione@enea.it; 4 Institute for Polymers, Composites and Biomaterials (IPCB), CNR, Via Campi Flegrei 34, 80078 Pozzuoli (Na), Italy; 5 National Interuniversity Consortium of Materials Science and Technology (INSTM), Piazza S. Marco, 4, 50121 Florence, Italy

**Keywords:** PEDOT:PSS, eumelanin, melanin, conducting polymer, bioinspired materials

## Abstract

The integration of the pristine not-doped commercial poly(3,4-ethylenedioxythiophene):poly(styrenesulfonate) (PEDOT:PSS) PH1000 with eumelanin, the brown to black kind of melanin pigment, was achieved by dissolving the melanogenic precursors 2-carboxy-5,6-dihydroxyindole (DHICA) in the PH1000 suspension. Solid state oxidative polymerization of the catecholic indole allowed obtaining the ternary blend PEDOT:PSS/eumelanin. The introduction of DHICA into PH1000 produced a noticeable increase in the conductivity of PEDOT thin films akin to that produced by dimethyl sulfoxide (DMSO) treatment, opening up novel strategies for the simultaneous integration of eumelanin polymer and conductivity enhancement of PEDOT containing coatings, as well as the long term goal of replacing PSS by DHICA eumelanin for PEDOT pairing.

## 1. Introduction

Organic electronics and the more recent organic bioelectronics are fast evolving technological sectors [1], which present several chief applications, in particular for the development and fabrication of active biointerfaces [2,3]. One of the key points, still to be fully addressed, to increase the availability of organic devices and applications on the market, is the development and true exploitation of a good conductive transparent organic material to replace indium tin oxide (ITO) [4]. In the course of the years, many materials have been proposed [5,6], but each of them exhibits at least one disadvantage. Just to name few of the many materials tested so far, poly(3,4-ethylenedioxythiophene):poly(styrenesulfonate) (PEDOT:PSS) is hygroscopic [7] and suffers immersion into the water [8]; graphene is not available for films with uniform and homogeneous properties on large area substrates [9]; polyaniline (PANI) conductivity is not as high as the applications request [10]; and dispersion of metallic nanoparticles or of carbon nanotubes (CNTs) inside conductive polymers can produce roughness [11], thick films [12], or reduced transparency [13].

Recent studies addressed the blending of PEDOT:PSS (the most used conductive organic material so far, because of its rather good conductivity, transparency, chemical stability, easy processing, and cost) with eumelanin precursor 5,6-dihydroxyindole (DHI) [14] to confer cohesion [15] and water resistance to PEDOT-based thin films. The new blend, named EuPH, still exhibited suitable conductivity to be employed as the anodic material within organic LEDs (OLEDs) [14]. The EuPH showed an overall increase of the crystalline order of the PEDOT associated with the approaching of the PEDOT chains at low eumelanin contents, but future lower conductivity and higher visible absorption than PH1000. It remains highly desirable to avoid any loss in terms of electrical performance and optical properties of eumelanin-based PEDOT blends.

In furthering the investigation of ternary PEDOT-eumelanin blends, we moved towards the other eumelanin precursor 2-carboxy-5,6-dihydroxyindole (DHICA) [16], which is known to afford eumelanins more transparent in the visible region than DHI-melanins. Moreover, the integration of anion-bearing polymers into PEDOT:PSS offers the perspective of the long term goal to replacing PSS by DHICA eumelanin as a PEDOT pairing agent.

In order to pair the eumelanin integration into the PEDOT:PSS, with affects possibly associated with PSS removal/segregation [15,17] such as conductivity enhancement, we devised to introduce a parallel poly-anionic melanin derivative into PEDOT:PSS, in order to possibly gain in terms of the PSS being replaced by the eumelanin. DHICA was used as the eumelanin precursor in the blends, and its oxidative polymerization was allowed to run in the solid phase after the films were prepared. The new material presented here, for sake of simplicity, is named as C-EuPH, or carboxylated EuPH.

As charge transport properties in PEDOT/PPS are strongly determined by the structural organization of both PEDOT and the poly anionic PSS at the molecular level, relevant enhancing of the PEDOT:PSS conductivity is obtained by so-called solvent doping, which involves the treatment of preformed films with polar solvents, typically DMSO [15,17,18]. This procedure is reported to produce a partial removal of the PSS and a general improvement of the PEDOT packing, finally resulting in a higher conductivity. Several protocols have been developed involving DMSO treatment of PEDOT:PSS [19], with the most suitable one to provide specific information about molecular reorganizations within the solid thin film seeming to be vapor exposition [19]. This protocol was adopted here to get insights into the relationship between DHICA content and blend conductivity.

## 2. Result and Discussion

**Scheme 1:**
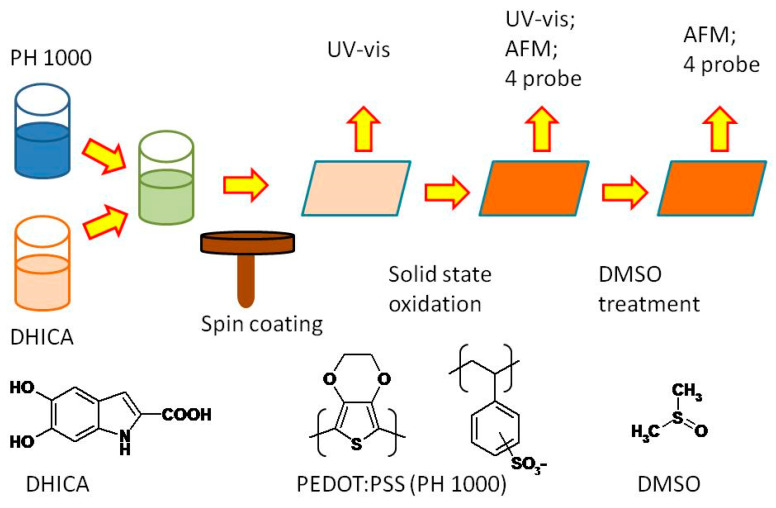
Key steps of the carboxylated EuPH (C-EuPH) preparation and characterization. AFM, atomic force microscopy; DHICA, 2-carboxy-5,6-dihydroxyindole; PEDOT:PSS, poly(3,4-ethylenedioxythiophene):poly(styrenesulfonate).

C-EuPH thin films were prepared (see Scheme 1) starting from mixtures (DHICA/PEDOT:PSS w/w ratios of X:1:2.5), obtained by combining a suitable solution of DHICA in isopropyl alcohol with a dispersion of PEDOT:PSS in water (the commercial Clevios™ PH 1000) [20]. For comparative purposes, films of sole DHICA-eumelanin, PSS, and PH-1000 were also prepared. All the films featured a thickness in the range of 400–1000 nm.

To allow straight characterization, the films were fabricated on quartz and glass substrates using spin coating deposition of the starting appropriate DHICA/PEDOT:PSS mixtures, and subsequent oxidative polymerization of DHICA in the solid state by the ammonia-induced solid state polymerization (AISSP) protocol.

Spectroscopic and electrical properties were determined for the films as formed and after standard “dedoping” protocols [15], in order to elucidate the possible DHICA contribution to the conductivities of the blends.

**Figure 1:**
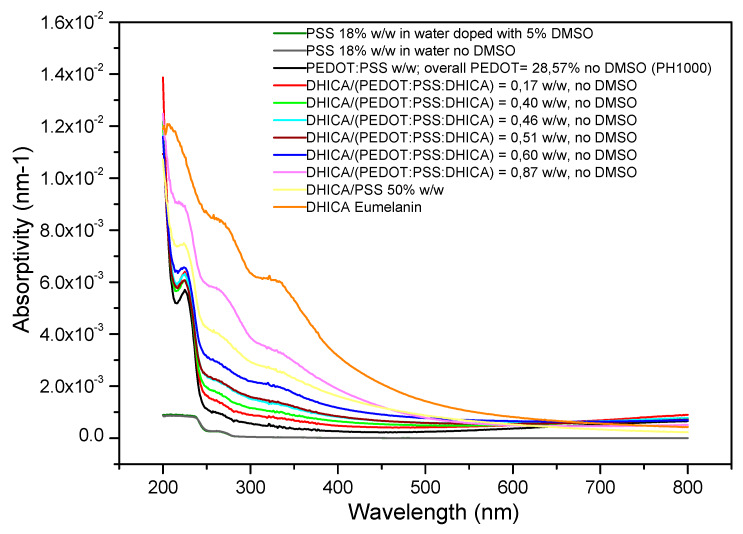
Absorptivity in the UV/vis range of carboxylated EuPH (C-EuPH) samples. Thickness of all the films is in the range of 400–1000 nm (Table S1). It is evident how the increase in the 2-carboxy-5,6-dihydroxyindole (DHICA)-eumelanin content increases the absorption in the UV region of the films. PEDOT:PSS, poly(3,4-ethylenedioxythiophene):poly(styrenesulfonate).

The effective conversion of DHICA into eumelanin pigment was assessed by UV/vis spectroscopy (Figure 1 and Figure S1). The various films of C-EuPH present the same ratio between PEDOT and PSS of 1:2.5, forced by the use of the PH1000, and different contents of DHICA in the starting mixtures, indicated in the figure legend as the weight ratio between DHICA and PEDOT. The percentages of the PEDOT in the mixtures, reported in the figure, are related to the weight of the PEDOT with respect to the total of the materials, that is, the ratio of PEDOT/(PEDOT + PSS + DHICA). Notably, because of the regiochemistry of the DHICA polymers, the absorption contribution of DHICA eumelanin in the visible range is well reduced with respect to DHI-based eumelanin pigments [16]. Indeed, the profiles in Figure 1 disclose the increase in the films’ absorptivity to be essentially confined below 400 nm, a feature of relevance for optoelectronic applications, owing to the formation of the DHICA-eumelanin in the films.

**Figure 2:**
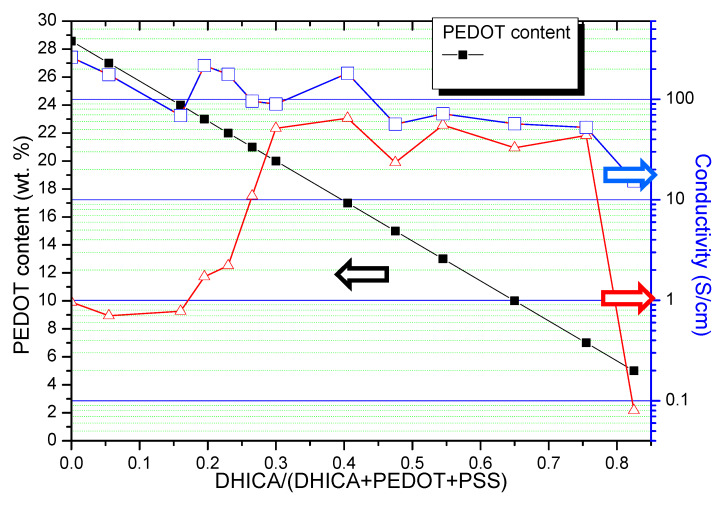
The electrical conductivity of the films featuring various DHICA contents (DHICA weight fractions DHICA/(PEDOT + PSS + DHICA): 0, 0.055, 0.160, 0.195, 0.230, 0.265, 0.300, 0.405, 0.475, 0.545, 0.650, 0.755, 0.825). Data were measured before (triangle) and after (open square) DMSO treatment. The black full squares indicates the content of the PEDOT respect to all the components of the film, that is, proportional to PEDOT/(DHICA + PEDOT + PSS).

The electrical conductivity of the films featuring the different DHICA content was evaluated by sheet-resistance measurements, performed with a four-point probe system, and the measured data are reported in Figure 2. As the increase of the PEDOT:PSS films’ conductivity is largely dependent upon post processing treatments, such as the exposition to polar solvent vapors [15], blank conductivity measurements were collected for the not-doped PEDOT:PSS thin films, as obtained after spin coating and annealing at 115 °C for 20 min, and then after exposition to DMSO vapors overnight at 100 °C. As prepared PH1000 had a conductivity around 1 S/cm, which rises to about 261.5 S/cm after the exposure to the DMSO, lower than that claimed by the manufacturer (a value of 850 S/cm according to Heraeus Clevios™ PH1000 Note #81076212), in the case of adding 5% of DMSO to the initial mixture prior to the preparation of the films (Heraeus Clevios™ PH1000 Note #81076212). This discrepancy was expected because of the lack of DMSO in the starting solution, but anyway, the obtained value is well within the range of the order of magnitude, thus warranting the solidity of the experimental protocol.

Conductivity values of the blends featuring DHICA weight content from 0% to 82.5% are reported in Figure 2, before (red line, triangles) and after (blue line, open squares) the treatment of the films with DMSO vapors.

As expected, films not treated with DMSO vapors feature a lower conductivity, although this difference is strongly dependent on DHICA content. In the absence of DHICA and for DHICA contents up to ca. 20%, the conductivity increase after the DMSO treatment is very large (two orders of magnitude and more); however, beyond ca. 25% of DHICA, the impact of the DMSO treatment is still evident, but the conductivity increase varies only from 20% more to about three times more. Indeed, it appears that the presence of the DHICA induces a marked conductivity increase in the films. Moreover, this effect is pretty regular over a quite wide range of DHICA content, despite the low conductivity of the DHICA melanin with respect to the PEDOT:PSS; therefore, a conductivity decay of the C-EuPH could be expected with the increase in DHICA content.

**Figure 3:**
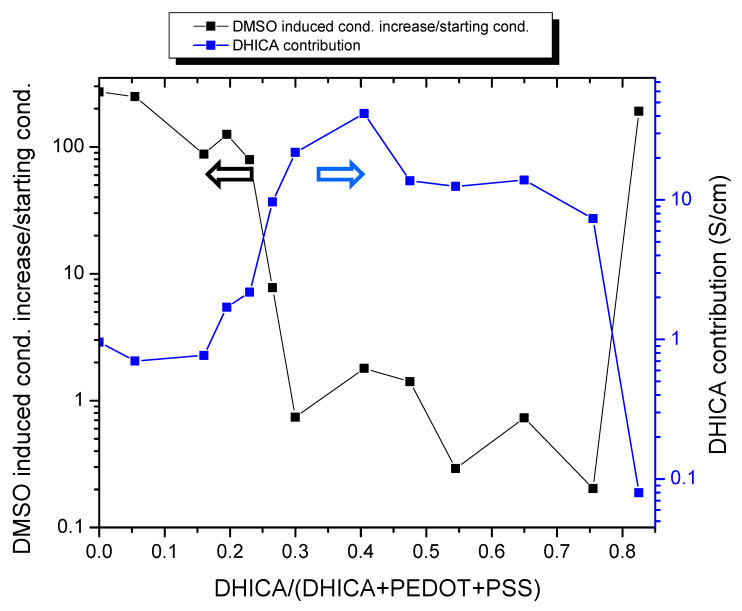
Conductivity increase in C-EuPH after DMSO treatment (left axis) and product of conductivity increase and the ratio of the conductivities before and after DMSO treatment (ΔCond × CondB/CondA).

It could be argued that the increased eumelanin content in C-EuPH could be responsible for a hardening of the films, reducing the motion freedom of the polymer chains, particularly the PSS, and thus limiting the impact of the DMSO on the molecular reorganization. This effect should be more pronounced after the increase of the eumelanin content in the C-EuPH. On the contrary, at a very high DHICA percent content, a marked conductivity increase can still be observed after the DMSO treatment of the films. At such high eumelanin contents, other effects could be produced by DMSO, beyond PSS segregation. If the conductivity of the C-EuPH is considered before the DMSO treatment (Figure 2, triangles), the presence of at least 20% of the DHICA-melanin already produces a marked increase in the conductivity of the films. This effect is detailed in Figure 3, reporting (left axis) the conductivity increase in the C-EuPH after the DMSO treatment normalized by the initial condition (conductivity after DMSO treatment-conductivity before DMSO treatment)/conductivity before DMSO treatment) as a function of DHICA content.

This plot highlights how the relative contribution of DMSO doping to the conductivity increase gets smaller as the DHICA content increases, up to a certain level. Actually, if we look at the absolute difference in conductivity before and after DMSO treatment (Figure S2), we observe its continuous decrease with DHICA content, while if we consider the ratio (Figure S2) between the two values, it follows a continuous increase with DHICA content. Indeed, DHICA produces some decrease of the higher achievable conductivity in the blend, but at the same time, it contributes to the increase of conductivity with respect to the not “dedoped” blend. The combination of these effects is reported in Figure 3 (right axis), where the product of conductivity increase and the ratio of the conductivities before and after DMSO treatment is reported. This last function can be considered an estimation of DHICA contribution to the increase of the conductivity of PEDOT with respect to the maximum increase for the given blend after DMSO treatment. In other words, if we consider both DHICA and DMSO as secondary doping agents, we estimate their contribution with respect to the overall possible increase, looking at that DHICA content, where the product of the absolute increase and relative increase is maximized.

The increase of the film conductivity because of the presence of the DHICA in the starting mixture was not observed for DHI [21] and could be, to some extent, owing to the presence of the carboxylic function in the DHICA indole, which is partially in the ionic form [22], and can produce electrostatic repulsion over PSS chains, with a consequent PSS-rich phase segregation and the associated increase in the conductivity of the films [23,24].

**Figure 4:**
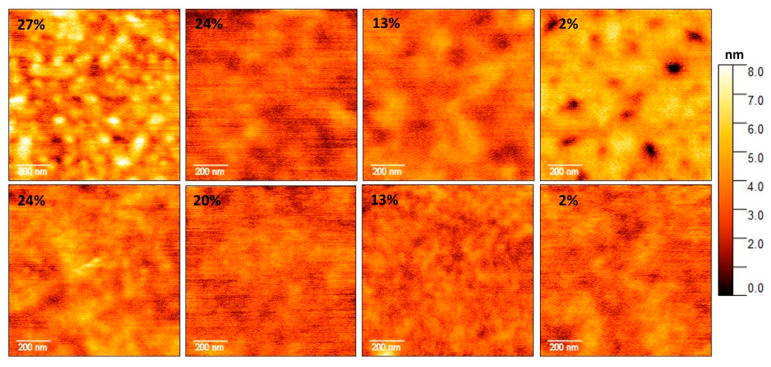
Atomic force microscopy (AFM) topography of the investigated C-EuPH samples. Top panels refer to the samples not treated with DMSO and bottom panels refer to those prepared in DMSO. The PEDOT weight percent is reported in the top-left corner of each image, DHICA weight percent is as follows: PEDOT 27%–DHICA 6%; PEDOT 24%–DHICA 16%; PEDOT 20%–DHICA 30%; and PEDOT 2%–DHICA 94%, respectively. Scans are 1 µm^2^ with a resolution of 256 × 256 pixels.

**Figure 5:**
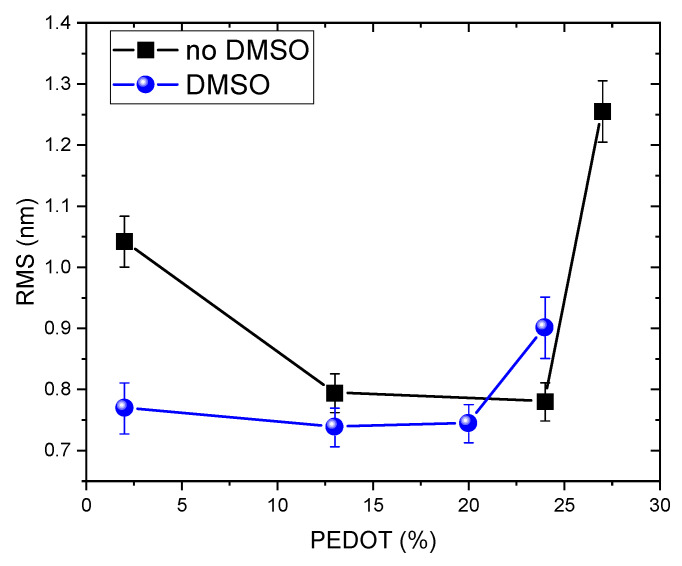
Estimated root means square (RMS) of the samples surface for non-DMSO- and DMSO-treated samples. Lower percentages of the PEDOT are the result of higher contents of DHICA.

To acquire additional insights supporting the possible involvement of DHICA and DHICA-eumelanin in driving segregation-like processes in PEDOT/PPS thin films, the morphology of C-EuPH thin film was investigated by atomic force microscopy (AFM). The film topography was investigated at a high resolution with a scan of 1 µm^2^ and a resolution of 4 nm/pixel, and the data are reported in Figure 4. From the measured AFM topography, we retrieve the height distribution curves (Figure S3) and the surface roughness estimated as the root mean square of the curves (Figure 5).

Notably, the presence of the DHICA-melanin is associated with a decrease of the surface roughness (Figure 4 top panels), going from the lowest DHICA-melanin content to the highest one. Deviation from this trend is only observed for the DHICA-rich (PEDOT-poor, less than 5% of PEDOT) samples, which can be explained by the formation of a more complex topography, presenting holes and wrinkles also observed in eumelanin thin films [25].

A qualitatively similar trend is also observed in the samples after the DMSO treatment, as evident by the comparison of the top and the bottom panels of Figure 4. In the region of 27%–20% of the PEDOT content, the DMSO treatment leads to an increase of the roughness, which has been previously observed [15,26] and likely ascribed to a PSS segregation effect and the formation of small grains of the PEDOT:PSS. As the PEDOT content decreases, DMSO seems have no effect on the roughness; for example, the treated and not-treated samples with a PEDOT content of 13% show the same roughness, within the experimental errors. This indicates that the roughness is mainly dominated by the presence of the eumelanin. In this respect, it is worth noting that this behavior is observed in the DHICA-melanin content range associated with the highest DHICA contribution to the conductivity (30%–40%; Figure 3).

Further support to possible segregation of PSS induced by the presence of DHICA-melanin was provided by the AFM phase imaging. In Figure S4, clear evolutions of the thin film morphologies are observed following both the increase of DHICA content in the blends as well as the DMSO treatment. Although the contribution of DHICA-melanin to the phase signal has yet to be defined, based on the literature, the bright and dark regions indicate the hard (PEDOT) and soft (PSS) microphases, respectively [15], so that the observed increase of their extensions is ascribable to the separation of PEDOT-rich domains.

## 3. Conclusions

To the best of our knowledge, very few studies are reported dealing with blending of PEDOT:PSS with other polymers [27,28,29], and even fewer exploiting some kind of polymer integration in the solid phase of a PEDOT:PSS layer [30]. The introduction of small molecules as well as polymers to PEDOT:PSS blends allows to get several achievements including conductivity enhancement, obtained by introducing small molecules such as sorbitol [31]; the increase of stretchability or adhesion by integrating plasticizers [27] or elastic polymers [27], respectively; or by means of DHI-derived melanins [14]. Each of these approaches offers advancements, but also suffers specific and common limitations. Examples of specific limitations are given by the leach of dopants, especially small molecules, or the loss of conductivity often observed for polymer dopants. The common limitation lays in the fact that each approach actually addresses only one issue: conductivity, adhesion, stretchability, and so on. Here, in the long-term perspective to achieve a multifunctional doping of PEDOT-based layers, the effect of the integration of the negatively charged DHICA-melanin within PEDOT:PSS layers was investigated, in terms of the impact on the blend conductivity, before and after the so-called “solvent dedoping” involving DMSO vapor treatment.

Notably, the presence of the DHICA-melanin in the films produced a relevant increase in the conductivity of the resulting non-doped carboxylated eumelanin PH1000 blends (here called C-EuPH), giving values comparable to those produced by the DMSO vapor treatment. Indeed, since the early observation by Kim et al. [32], the enhancement of the conductivity in PEDOT:PSS thin films achieved by treatment with DMSO or with dimethylformamide (DMF) is associated with the PSS-rich phase segregation [23,24,33]. The integrated spectroscopic and morphological investigations reported here strongly suggest that the blending of the DHICA-melanin into the PEDOT:PSS also succeeds in performing PSS segregation, with a resulting increase of the films’ conductivity.

Although further investigations are required to definitively link the dependence of the C-EuPH conductivity on the DHICA-melanin content, with the associated effects on the PSS induced by the DHICA-melanin itself, it may be speculated that the anionic function of the DHICA polymeric chains can partially substitute one of the PSS in the molecular organization of the blends. Moreover, the introduction of the DHICA eumelanin confers a lower absorption in the visible spectrum with respect to that of EuPH [16], expanding the scope of the eumelanin-PEDOT blending to design and fabricate optoelectronic devices.

## 4. Experimental Section

### 4.1. Materials and Methods

All commercially available reagents were used as received and all the solvents were analytical grade quality. More details concerning materials and methods are reported in the Supporting Information.

### 4.2. C-EuPH Thin Film Fabrication

All commercially available reagents were used as received and all of the solvents were analytical grade quality. Deionized water (resistivity was 18 MΩ·cm).

The C-EuPH blend was prepared by mixing, under inert atmosphere, equal volumes of two mother solutions: (a) DHICA in isopropyl alcohol (8 mg/mL) and (b) PEDOT:PSS dispersion in water (Heraeus Clevios™ PH1000 (Hanau, Germany) commercial product, featuring ca. 1.3% weight content of polymers, with a PEDOT:PSS ratio of 1:2.5).

The DHICA-PEDOT:PSS blend was filtered through a 0.45 μm Whatman membrane (Maidstone, UK) and its films were deposited by spin coating on glass substrates (Corning Eagle XG (New York, NY, USA), previously cleaned by sonication in deionized water and UV-ozone treated) using a Laurell Technologies Corp. WS-650MZ-23NPP/LITE spin coater (North Wales, PA, USA). Films were annealed at 80 °C for 30 min on a hot plate in the inert atmosphere.

The C-EuPH thin films were obtained by exposing the DHICA-PEDOT:PSS films for 1 h to air-equilibrated gaseous ammonia, from an ammonia solution (28% in water) inside a sealed chamber at 1 atm pressure and at a controlled temperature (c.a. 25 °C).

### 4.3. Materials’ Characterizations

UV/vis spectra were acquired using a Perkin-Elmer Lambda 900 spectrophotometer (Waltham, MA, USA).

Sheet resistance measurements were performed using a four-point probe system Napson RESISTAGE RG-80 (Tokyo, Japan).

Thickness of the films was measured using a stylus profilometer KLA Tencor P-10 (Milpitas, CA, USA).

AFM images were observed through an Atomic Force Microscope WITec Alpha 300 RAS (Ulm, Germany), working in tapping mode with non-contact cantilevers at a resonance frequency of 280 kHz and spring constants of k = 42 N/m. All of the analyzed AFM images have dimensions of 1 × 1 µm^2^, with 256 points per scan line and 256 scan lines, and at a 1 Hz scan rate (i.e., at a corresponding tip to sample velocity of 1 µm/s).

## Supplementary Materials

The following are available online at https://www.mdpi.com/1996-1944/13/9/2108/s1, Figure S1: Comparison of the UV/vis profiles of DHICA, DHICA-melanin, PH100, and C-EuPH, and evolution of the UV/vis profile of DHICA after oxidation to DHICA melanin. Arrows denote the trends; yellow region is the one witnessing the completeness of DHICA oxidation within C-EuPH; Figure S2: Blend conductivity difference before and after DMSO treatment ◊ (right axis) and the ratio □ (left axis) between the two values vs. DHICA content; Figure S3: Height distribution curves retrieved from the AFM scans. Each curve is plotted in order to obtain its mean value at Z = 0. The calculated RMS represents the roughness of the sample surface; Figure S4: AFM topography of selected C-EuPH samples: (1) PEDOT 13%–DHICA 64%; (2) PEDOT 13%–DHICA 64%, phase mode; (3) PEDOT 24%–DHICA 16%, phase mode; (4) PEDOT 13%–DHICA 64%, DMSO-treated; (5) PEDOT 13%–DHICA 64%, DMSO-treated phase mode; Table S1: Thickness of the different films measured; Methods.
