# Supplementary material for: Eumelanin Precursor 2-Carboxy-5,6-Dihydroxyindole (DHICA) as Doping Factor in Ternary (PEDOT:PSS/Eumelanin) Thin Films for Conductivity Enhancement
Source: Materials (Basel). 2020 May 2;13(9):2108. doi: 10.3390/ma13092108 (PMC7254328; doi:10.3390/ma13092108)
Supplement: Supplementary file 1 [file materials-13-02108-s001.pdf]

materials

MDPI

Supplementary

Eumelanin Precursor 2-Carboxy-5,6-Dihydroxyindole (DHICA) as Doping Factor in Ternary (PEDOT:PSS:Eumelanin) Thin Films for Conductivity Enhancement

Ludovico Migliaccio [1,†], Felice Gesuele [2,†], Paola Manini [1], Maria Grazia Maglione [3], Paolo Tassini [3,*], and Alessandro Pezzella [4,5,*]

1 Department of Chemical Sciences, University of Naples "Federico II", 80126 Naples, Italy; ludovico.migliaccio@unina.it (L.M.); paola.manini@unina.it (P.M.)
2 Department of Physics "Ettore Pancini", University of Naples "Federico II", 80126 Naples, Italy; felice.gesuele@unina.it
3 Laboratory for Nanomaterials and Devices (SSPT-PROMAS-NANO), ENEA C. R. Portici, Piazzale Enrico Fermi 1, Località Granatello, 80055 Portici, Italy; mariagrazia.maglione@enea.it (M.G.M.); paolo.tassini@enea.it (P.T.)
4 Institute for Polymers, Composites and Biomaterials (IPCB), CNR, Via Campi Flegrei 34, 80078 Pozzuoli (Na), Italy
5 National Interuniversity Consortium of Materials Science and Technology (INSTM), Piazza S. Marco, 4, 50121 Florence, Italy
† These authors contributed equally
* Correspondence: paolo.tassini@enea.it (P.T.); alessandro.pezzella@unina.it (A.P.)

Received: 8 April 2020; Accepted: 29 April 2020; Published: date

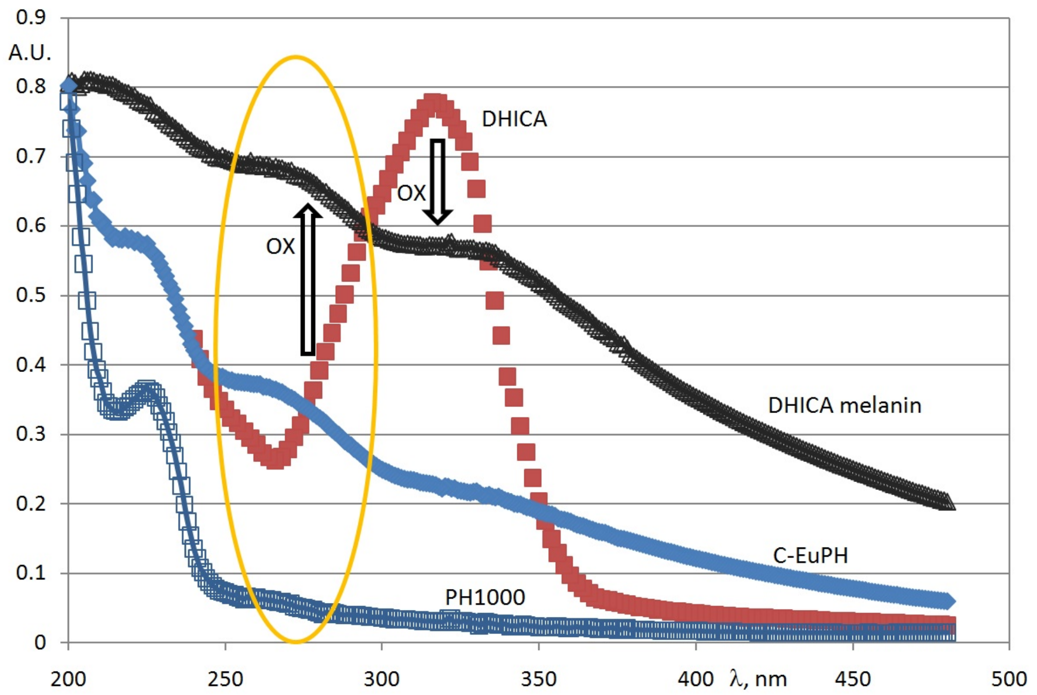

Figure S1. Comparison of the UV/vis profiles of DHICA, DHICA-melanin, PH100, and C-EuPH, and evolution of the UV/vis profile of DHICA after oxidation to DHICA melanin. Arrows denote the trends; the yellow region is the one witnessing the completeness of DHICA oxidation within C-EuPH.

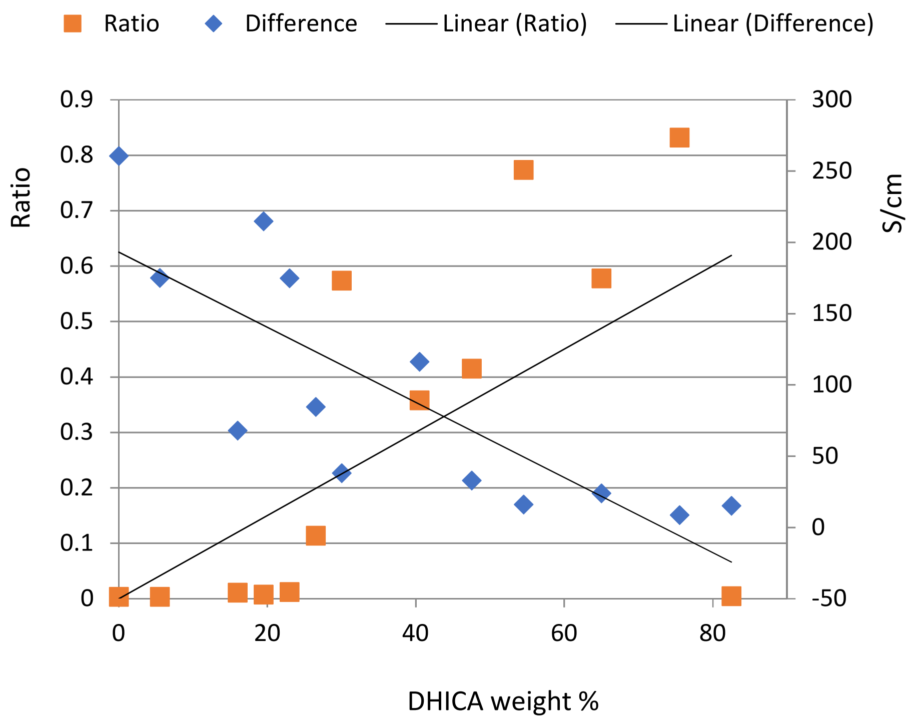

Figure S2. Blend conductivity difference before and after DMSO treatment ◊ (right axis) and the ratio □ (left axis) between the two values vs. DHICA content.

Table 1. Thickness of the different films measured. DHICA, 2-carboxy-5,6-dihydroxyindole; PEDOT:PSS, poly(3,4-ethylenedioxythiophene):poly(styrenesulfonate).

Sample	Thickness (nm) +/−5
PEDOT/PSS no DHICA	79
DHICA 0.17 w/w	84
DHICA 0.40 w/w	90
DHICA 0.46 w/w	96
DHICA 0.51 w/w	112
DHICA 0.60 w/w	100
DHICA 0.76 w/w	160
DHICA 0.87 w/w	114
DHICA 0.94 w/w	95
DHICA	60

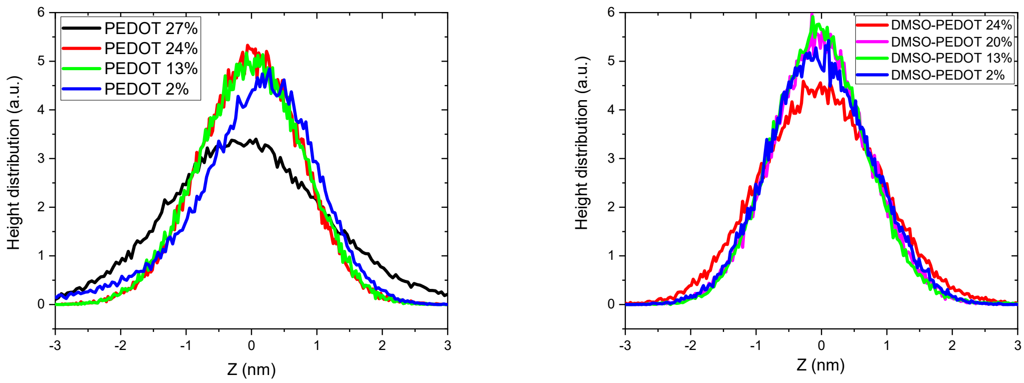

Figure S3. Height distribution curves retrieved from the AFM scans. Each curve is plotted in order to obtain its mean value at Z = 0. The calculated RMS represents the roughness of the sample surface.

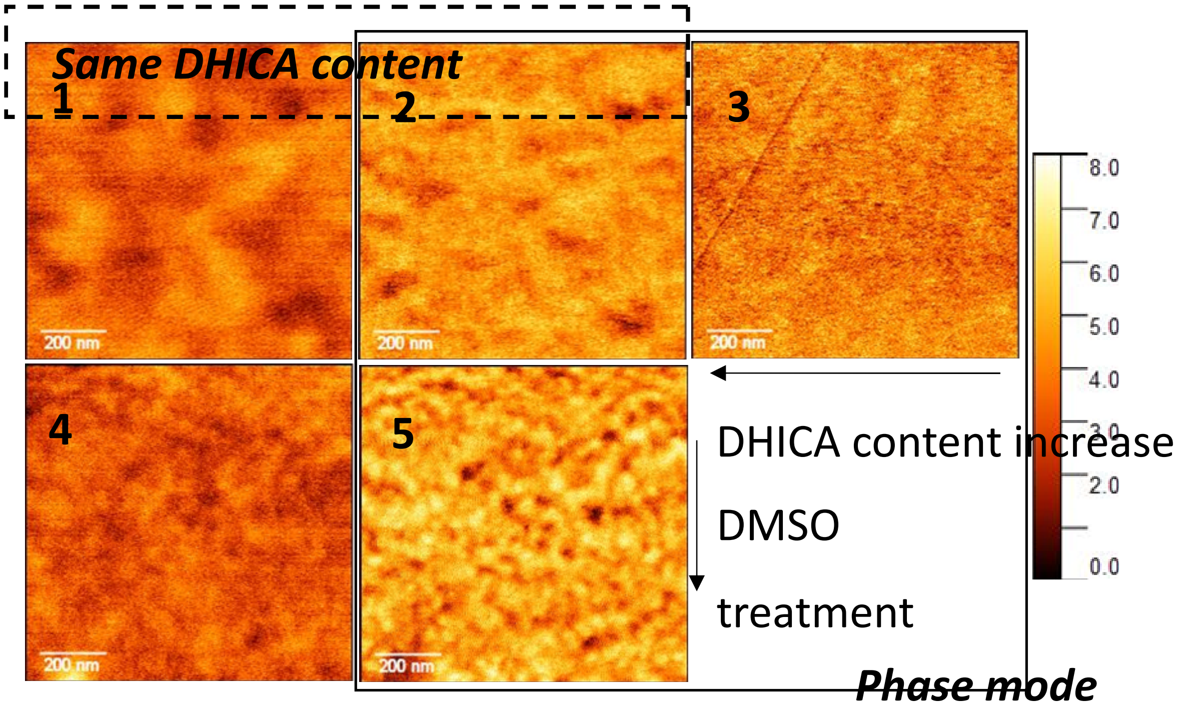

Figure S4. AFM topography of selected C-EuPH samples: (1) PEDOT 13%–DHICA 64%; (2) PEDOT 13%–DHICA 64%, phase mode; (3) PEDOT 24%–DHICA 16%, phase mode; (4) PEDOT 13%–DHICA 64%, DMSO-treated; and (5) PEDOT 13%–DHICA 64%, DMSO-treated phase mode.

Methods

Materials and Methods

All commercially available reagents were used as received and all of the solvents were analytical grade quality.

2-carboxy-5,6-dihydroxyindole (DHICA) was prepared according to a reported procedure [1].

Morphological roughness images were taken by means of The Taylor Hobson® CCI HD non contact 3D Optical Profiler with thin and thick film measurement capability. It uses an innovative, patented correlation algorithm to find the coherence peak and phase position of an interference

pattern produced by our precision optical scanning unit. The new CCI HD merges world leading non-contact dimensional measurement capability with advanced thin and thick film technology.

The CCI HD was designed to offer both types of film thickness measurement in addition to dimensional and roughness capability.

The film thicknesses were measured by KLA Tencor P-10 surface profiler. Film thickness was estimated by scratching the film down to the substrate with a needle and measuring by surface profiler the height of the resulting trench.

Film roughness was estimated as root mean square (RMS) from several scans on the same sample.

UV/vis optical transmittance analysis of layers deposited on glass substrates was carried out by using Perkin-Elmer Lambda 900 spectrophotometer.

DHICA and C-EuPH Thin Film Fabrication

DHICA and C-EuPH thin films were prepared by spin coating appropriate solutions with a Laurell WS-650MZ-23NPP/LITE coater; thin films were deposited on glass substrates. Thin films were obtained using the following spin coating recipe: 600 rpm/s, 1100 rpm, and 60″.

The resulting films' thicknesses, measured by a KLA Tencor P-10 surface profiler, were in the range of 100–150 nm.

Ammonia-Induced Solid State Polymerization

The oxidation of DHICA and DHICA-PH1000 thin films (100–300 nm thickness), to give the melanin polymer, was achieved by exposure to an oxidizing atmosphere (e.g., oxygen atmosphere and ammonia vapors) [2]. In the general procedure, the appropriate film was incubated in the oxygen/ammonia atmosphere at a controlled temperature (25–40 °C). The ammonia vapors were produced by equilibration of the atmosphere with ammonia solution (28% to 7% NH_3 in H_2O) in a sealed camera at 1 ATM pressure for 1 h. When appropriate, the whole spin coating procedure was conducted under oxidation promoting atmosphere.

Oxidative polymerization was followed by UV/vis spectroscopy.

The resulting films' thicknesses, measured by a KLA Tencor P-10 surface profiler, were in the range of 120–130 nm.

DMSO Treatment

Treatment of the films with DMSO vapor was achieved placing the films in a chamber with DMSO vapors overnight equilibrated at 100 °C.

Electrical Characterization

The electrical conductivity of the films was evaluated from sheet-resistance measurements by means of a four-point probe system (Napson Co.).

For each C-EuPH and different DHICA contents, three samples were produced and each was measured in six different points for the evaluation of the sheet resistance using the four-point resist test tool. The sheet resistance value (average of the values) was multiplied by the thickness and the reciprocal gives us the conductivity value.

Atomic Force Microscopy

The topography of the samples was obtained by means of an atomic force microscope (AFM—Witec Alpha 300 RAS) working in tapping mode with non-contact cantilevers at resonance frequency of 280 kHz and spring constants of k = 42 N/m. All the analyzed AFM images have dimensions of 1 × 1 μm^2 with 256 points per scan line and 256 scan lines and at a 1 Hz scan rate (i.e., at a corresponding tip to sample velocity of 1 μm/s).

The resulting pixel size is 4 nm, which is equal or less than the tip dimensions (nominal radius of curvature R ≤ 10 nm).

The AFM parameters (non-contact operation mode, gain values, set-point value, scan rate, driving amplitude) were kept constant during the scans in order to ensure the roughness comparison among the samples.

The samples were analyzed in random order to eliminate nuisance factors resulting from the tip degradation or laboratory temperature and humidity variation.

Acquired images were analyzed with Gwyddion 2.51 (http://gwyddion.net/). Typical operations include baseline removal, plane subtraction, and match-line correction with median of differences. Roughness was estimated by extracting the distribution of the height values and calculating the root mean square (RMS) of the height irregularities from the second central moment of data values:

$$RMS = \sqrt{\frac{1}{N}\sum_{1}^{N}(z_n - \bar{z})^2} \quad ; \ \bar{z} \ denotes\ the\ mean\ value$$

Reference

1. Edge, R.; D'Ischia, M.; Land, E.J.; Napolitano, A.; Navaratnam, S.; Panzella, L.; Pezzella, A.; Ramsden, C.A.; Riley, P.A. Dopaquinone redox exchange with dihydroxyindole and dihydroxyindole carboxylic acid. *Pigm. Cell Res.* **2006**, *19*, 443–450.
2. Pezzella, A.; Barra, M.; Musto, A.; Navarra, A.; Alfè, M.; Manini, P.; Parisi, S.; Cassinese, A.; Criscuolo, V.; D'Ischia, M. Stem cell-compatible eumelanin biointerface fabricated by chemically controlled solid state polymerization. *Mater. Horiz.* **2015**, *2*, 212–220.
